# DINIES: drug–target interaction network inference engine based on supervised analysis

**DOI:** 10.1093/nar/gku337

**Published:** 2014-05-16

**Authors:** Yoshihiro Yamanishi, Masaaki Kotera, Yuki Moriya, Ryusuke Sawada, Minoru Kanehisa, Susumu Goto

**Affiliations:** 1 Division of System Cohort, Medical Institute of Bioregulation, Kyushu University, 3-1-1 Maidashi, Higashi-ku, Fukuoka 812-8582, Japan; 2 Institute for Advanced Study, Kyushu University, 6-10-1 Hakozaki, Higashi-ku, Fukuoka 812-8581, Japan; 3 Graduate School of Bioscience and Biotechnology, Tokyo Institute of Technology, 2-12-1 Ookayama, Meguro-ku, Tokyo, 152-8550, Japan; 4 Bioinformatics Center, Institute for Chemical Research, Kyoto University, Uji, Kyoto 611-0011, Japan

## Abstract

DINIES (drug–target interaction network inference engine based on supervised analysis) is a web server for predicting unknown drug–target interaction networks from various types of biological data (e.g. chemical structures, drug side effects, amino acid sequences and protein domains) in the framework of supervised network inference. The originality of DINIES lies in prediction with state-of-the-art machine learning methods, in the integration of heterogeneous biological data and in compatibility with the KEGG database. The DINIES server accepts any ‘profiles’ or precalculated similarity matrices (or ‘kernels’) of drugs and target proteins in tab-delimited file format. When a training data set is submitted to learn a predictive model, users can select either known interaction information in the KEGG DRUG database or their own interaction data. The user can also select an algorithm for supervised network inference, select various parameters in the method and specify weights for heterogeneous data integration. The server can provide integrative analyses with useful components in KEGG, such as biological pathways, functional hierarchy and human diseases. DINIES (http://www.genome.jp/tools/dinies/) is publicly available as one of the genome analysis tools in GenomeNet.

## INTRODUCTION

The identification of drug–target interactions, which are defined as interactions between drugs (or drug candidate compounds) and target proteins (or target candidate proteins), is an important part of genomic drug discovery. Several public databases have been established to store drug–target interactions, including DrugBank (1), Matador (2), STITCH (3) and KEGG DRUG (4), but most of the drug–target interaction network remains undiscovered. Recent developments in biotechnology have contributed to the increase in the amounts of high-throughput data for compounds and proteins in the genome, transcriptome, proteome, metabolome and phenome, which can be useful sources for inferring unknown drug–target interaction networks on a large scale. In this context, prediction methods of drug–target interactions, using all available omics data and other experiments, should be made more easily accessible to biologists in academic fields and the pharmaceutical industry to improve their research productivity.

A variety of computational methods have been developed for predicting drug–target interactions, or more generally compound–protein interactions, in the context of chemogenomics (5–10). Recently, the use of pharmacological data for drugs (e.g. pharmaceutical effects, side effects) has been proposed in the context of pharmacogenomics (11–14). There are web servers that implement some of these methods. For example, CDRUG is a web server used for predicting anticancer activity from chemical structures of compounds encoded by the Daylight fingerprint (15), and COPICAT is a web service for predicting compound–protein interactions from chemical structures of compounds and amino acid triplet frequencies of proteins (16). However, these existing servers have limitations with respect to the flexibility of the input data and biological interpretability of the prediction results.

In this study, we present drug–target interaction network inference engine based on supervised analysis (DINIES; http://www.genome.jp/tools/dinies/), a web server for predicting unknown drug–target interaction networks from various types of biological data (e.g. chemical structures, drug side effects, amino acid sequences and protein domains) in the framework of supervised network inference. The prediction is performed using state-of-the-art machine learning methods in chemogenomics and pharmacogenomics, assuming that similar compounds (not necessarily in chemical structures but in side effect profiles and other features) are likely to interact with similar proteins. This method is suitable for predicting potential off-targets of marketed drugs with known targets, and potential target profiles of new drug candidate compounds without known targets. The algorithms in DINIES have been previously published (6,12,14), and this web server represents the first public resource that implements these methods. The server is compatible with the KEGG database (4) by sharing the same identifiers, a feature that allows integrative analyses with useful components in KEGG, such as biological pathways, functional hierarchy, human diseases and drug classification.

## RATIONALE AND IMPLEMENTATION

### Data integration

**Figure 1.**
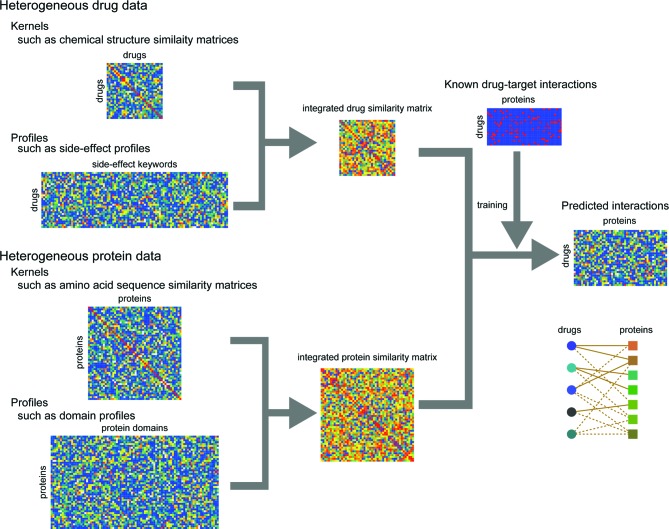
Overview of DINIES. DINIES accepts as inputs tab-delimited text files that must be in the forms of either a ‘profile’ or a ‘kernel matrix’ (a kernel, in short). We use ‘profile’ to denote an asymmetric matrix in which rows correspond to the objects of interest (drugs or proteins) and columns to the properties of the objects (such as chemical substructures and domains). We use ‘kernel’ to denote a symmetric matrix where rows and columns both correspond to objects (drugs or proteins).

Figure 1 shows an overview of DINIES, which accepts any ‘profiles’ of drugs (or drug candidate compounds) and target proteins (or target candidate proteins) (e.g. chemical fingerprints, drug side effect profiles, protein domain profiles) or precalculated similarity matrices of drugs and target proteins (e.g. chemical structure or amino acid sequence similarity matrices) in a tab-delimited file format. In DINIES, each data set describing drugs or proteins is transformed into a kernel similarity matrix (e.g. a correlation coefficient matrix) using a kernel function, where each element in the matrix corresponds to a drug–drug similarity or protein–protein similarity. Multiple similarity matrices generated from heterogeneous data sets are integrated into a single matrix using a linear combination of the similarity matrices (the sum of the identical-weighted matrices as default), which gives an integrated similarity matrix representing drug–drug or protein–protein similarities.

### Supervised network inference

Supervised network inference for drug–target interaction prediction involves two processes: a training process in which a predictive model is learned by exploiting the partial knowledge of the interaction network and a test process in which new drug–target interactions are inferred. Drug–protein pairs are predicted to interact whenever the prediction scores for the pairs exceed a threshold. There are several algorithms for learning an appropriate predictive model in the training process, including bipartite graph inference (6), pair-wise support vector machine (SVM) (5,7,8), bipartite local model (10) and pair-wise kernel regression (14). Three algorithms are implemented in DINIES, but the SVM-based methods are not implemented, owing to the prohibitive computational cost and to large memory consumption in the on-demand training phase. The detailed explanations about each method are written in the help page. Pair-wise kernel regression is the default algorithm in DINIES because of its computational efficiency, but users can select other algorithms.

## USER INTERFACE AND BASIC FUNCTIONS

The DINIES server provides two options: DINIES Search and DINIES Prediction.

### DINIES Search

The user can explore precalculated drug–target interaction networks that were predicted with available data in KEGG or other databases. In this case, the server accepts KEGG drug ID, drug name, MOL file text, and SMILES string. The target protein can be input using KEGG protein ID (e.g. hsa:4988). If the input compound is not present in the drug–target interaction network, the user can search for structurally similar drugs in the drug–target interaction network using a SIMCOMP chemical structure similarity search (17); however, this similarity search is not involved in the prediction. The predicted drug–target interaction network is prepared in advance by either a chemogenomic approach based on the precomputed chemical structure similarity of drugs or a pharmacogenomic approach based on the precomputed side effect similarity of drugs. Predictive models in chemogenomic and pharmacogenomic approaches are trained on known drug–target interactions not only in KEGG but also in other compound–protein interaction databases. The prediction results are available in the latest version of DINIES Search. The input threshold determines the minimum predictive value for the display of predicted drug–protein interactions.

### DINIES Prediction

The possible inputs of DINIES Prediction are any data sets about drugs and proteins that are represented as text files in the form of either a tab-delimited profile matrix or a kernel similarity matrix predefined by the user. For example, suppose we are given two profile matrices for drugs: chemical fingerprints and side effect profiles. Chemical fingerprints (or chemical descriptors) can be regarded as a binary (or real-valued) profile matrix in which rows represent drugs and columns represent the presence/absence (or numbers) of various chemical substructures. Side effect profiles can also be regarded as a binary (or real-valued) profile matrix in which rows represent drugs and columns represent the presence/absence (or numbers) of previously reported side effects (such as hyperthermia, gastrointestinal bleed and hepatic dysfunction). Examples of profile matrices for proteins are domain composition profiles and gene expression profiles. Domain composition profiles can be designed as a binary profile matrix in which rows represent proteins and columns represent the presence/absence of various domains. Gene expression profiles can be regarded as a real-valued profile matrix in which rows represent proteins or genes and columns represent expression levels in the respective experiments. Examples of similarity matrices for drugs include 2D chemical structure similarity scores and graph kernel similarity scores (18). Examples of similarity matrices for proteins include Smith–Waterman scores (19) and sequence kernel similarity scores (20). This flexibility is one of the strength of DINIES. If KEGG IDs are used for drugs or/and human proteins in the input data, drugs and proteins can be mapped onto many useful components in the KEGG database, such as biological pathways in KEGG PATHWAY and functional hierarchies in KEGG BRITE. Examples of input data files can be seen on the help page (http://www.genome.jp/tools/dinies/help.html).

In DINIES Prediction, there are two possible modes, ‘Simple mode’ and ‘Advanced mode’. Simple mode is provided for the users who choose to obtain the results with the default settings. In the simple mode, profile matrices are converted into kernel similarity matrices by linear kernel, and all kernels are integrated with the same weight. Furthermore, supervised learning by pair-wise kernel regression is performed using known drug–protein interactions in KEGG DRUG as training data. After the prediction result is obtained, the details of the default settings can be checked and modified to perform the prediction again with different parameters.

In advanced mode, users can choose one of the algorithms, kernel functions, training interaction data and some parameters in the method. In the default settings, known drug–protein interactions registered in KEGG DRUG are used as training data, although users also can use their own drug–protein interactions.

The computational cost depends on the numbers of drugs and target proteins in the training drug–target interaction data; it takes a few minutes to perform both training and prediction tasks, for example, when the training data consist of 600 drugs and 300 target proteins. If the calculation would require a long time, the users can select the e-mail option and be able to receive an e-mail notification when the calculation is complete.

### Output of DINIES

**Figure 2.**
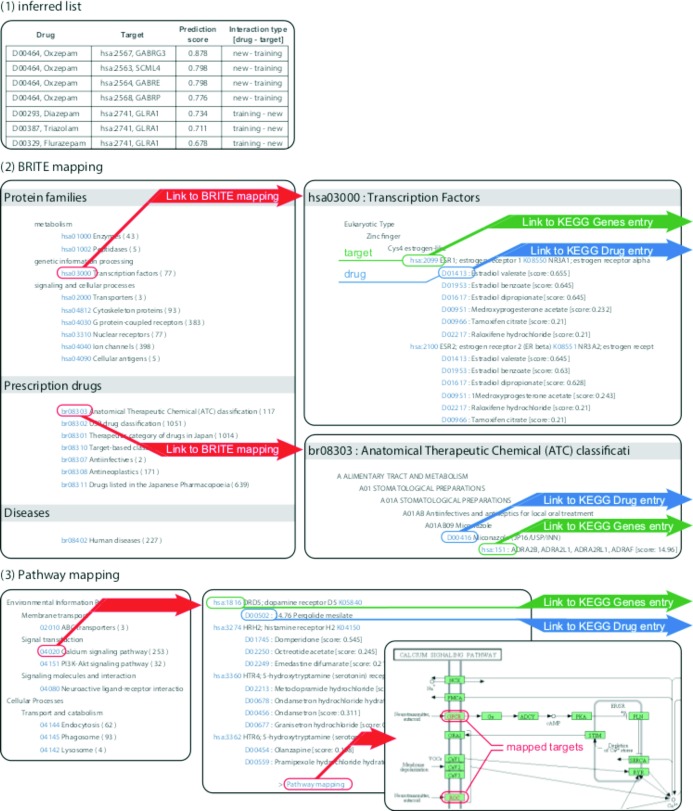
Output example of DINIES. (1) Inferred list classifies the drug–target interaction network into training–new, prediction–prediction and new–training, where ‘training’ and ‘new’ denote drugs/targets found and not found in the training data (KEGG DRUG in the default settings), respectively. Text files with the same format can be downloaded using the Download option. (2) The BRITE mapping option enables the user to find drugs or proteins of interest from the functional hierarchy defined in the KEGG BRITE database. (3) The Pathway mapping option shows the predicted drug–target interactions grouped on the basis of KEGG PATHWAY maps.

The output of DINIES, for either DINIES Search or DINIES Prediction, is a weighted bipartite graph with drugs and proteins as nodes and prediction scores for drug–protein pairs as edges. The prediction results are provided in the following ways (Figure 2): Inferred list, BRITE mapping, Pathway mapping and Downloadable text files. An example of the output can be observed at http://www.genome.jp/tools-bin/dinies?mode=path&id=example. The first option, Inferred list, provides the predicted interaction pairs categorized into (i) new drugs versus new proteins, (ii) training drugs versus new proteins, (iii) new drugs versus training proteins, (iv) training drugs versus training proteins and (v) all (including all predicted interactions and known interactions), where ‘training’ and ‘new’ correspond, respectively, to presence and absence in the training interaction data. Each drug–protein pair is assigned a prediction score. Higher score (closer to 1) can be interpreted more reliable (high confidence), lower score (closer to 0) can be interpreted less reliable (low confidence). The second option, BRITE mapping, outputs the predicted interactions grouped into many functional hierarchies in KEGG BRITE (e.g. protein families, drug classes and human diseases). When the user selects one of the protein families, the drugs that are predicted to interact with proteins in the selected family will be highlighted. The third option, Pathway mapping, outputs the predicted interactions grouped into biological pathway maps in KEGG PATHWAY (e.g. signalling pathways, metabolic pathways). When the user selects one of the pathways, proteins that are predicted to interact with drugs in the selected pathway will be highlighted. The last option, Download, provides a list of predicted drug–protein pairs downloadable as a tab-delimited text file, which can be viewed with network-visualizing software, such as Cytoscape (http://www.cytoscape.org/) (21).

## APPLICATION AND PERFORMANCE EVALUATION

The validity of supervised network inference algorithms for drug–target interaction prediction from single data sets for drugs or proteins has been shown in many previous studies by our groups and others (5–8,10,12,14). Here we show an application of DINIES to the integration of multiple heterogeneous data: two drug-related data sets, i.e. 2D chemical structure similarity (18) and Food and Drug Administration (FDA) side effect profiles (14) and two target-related data sets, i.e. protein amino acid sequence similarity (20) and PFAM domain composition profiles (22).

First, we performed a large-scale prediction of the unknown drug–target interaction network for 4227 drugs and 471 target proteins using the DINIES system based on learning with known drug–target interactions in KEGG DRUG. When the prediction score threshold was set to 0.5, the DINIES system predicted 733 drug–protein pairs as potential interacting pairs. We investigated the newly predicted drug–target pairs (absent from KEGG DRUG) using the other compound–protein interaction databases DrugBank (1), Matador (2), ChEMBL (23), PDSP Ki (24) and TTD (25). We were able to confirm the validity of 84 pairs with DrugBank, 23 pairs with Matador, 70 pairs with ChEMBL, 17 pairs with PDSP Ki and 24 pairs with TTD. The detailed list of confirmed drug–protein pairs can be seen in the supplemental materials on the help page. These results suggest the reasonable performance of DINIES. There remain many unconfirmed drug–target pairs, so experimental validation of the other unconfirmed drug–target pairs would be an important future task.

**Figure 3.**
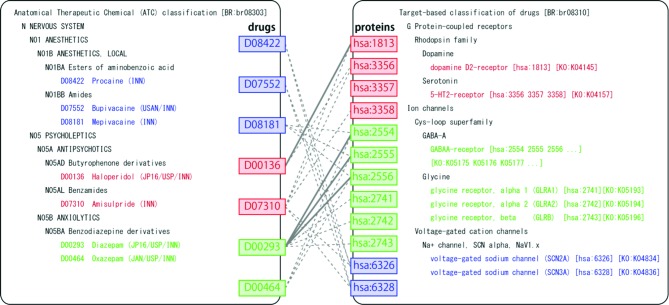
BRITE mapping of the predicted drug–target interactions. Left and right panels show BRITE functional hierarchies for drugs and human proteins, respectively. Solid and dotted gray lines represent known and predicted drug–protein interactions, respectively.

Figure 3 shows examples of the predicted drug–target interactions mapped onto KEGG BRITE, which illustrates the association between drug Anatomical Therapeutic Chemical (ATC) classification and protein family hierarchy in terms of predicted drug–target interactions. For example, Diazepam (D00293), a sedative–hypnotic drug, was predicted to interact with GLRA1 (glycine receptor, alpha 1) [hsa:2741], GLRA2 (glycine receptor, alpha 2) [hsa:2742] and GLRB (glycine receptor, beta) [hsa:2743]. These predictions were confirmed in Matador (2). Bupivacaine (D07552), an anesthetic drug, was predicted to interact with SCN2A (voltage-gated sodium channel, type II) [has:6326] and SCN3A (voltage-gated sodium channel, type III) [has:6328]. Mepivacaine (D08181), an anesthetic drug, was also predicted to interact with SCN2A and SCN3A. These predictions were confirmed in ChEMBLE (23). As observed above, DINIES provides comprehensive prediction of drug–protein interactions based on various heterogeneous inputs. Furthermore, mapping predicted drug–target pairs onto KEGG BRITE and KEGG PATHWAY may provide us with biological understanding of drug effects at the level of molecular interaction networks. Such knowledge could be useful for avoiding possible side effects or discovering new effects of existing drugs for different therapies (drug repositioning). In practice, users may want to know the specificity of the drug–target interaction of interest to design a novel drug that binds specifically to the target. DINIES does not provide direct evidence but provides clues, such as the number of predicted interaction partners for the drugs/proteins of interest.

In addition, we evaluated the performance of DINIES by cross-validation experiments. In benchmark data construction, we removed very similar drugs, sharing a chemical structure similarity of 0.8 or above, and very similar proteins, sharing a sequence similarity of 0.8 or above, to avoid overestimating prediction accuracy because of data duplication or close homologs. We obtained benchmark data consisting of 678 diverse drugs, 277 diverse proteins and their 1804 interactions. We assume primarily two practical situations: (i) detection of missing interactions between existing drugs and known target proteins and (ii) prediction of potential interactions involving newly discovered drug candidate compounds and newly discovered target candidate proteins. From these two viewpoints, we performed two types of 3-fold cross-validation: pair-wise and block-wise cross-validation. In the pair-wise cross-validation, drug–target pairs in the benchmark data were randomly split into a training set and a test set. In the block-wise cross-validation, drugs (resp. target proteins) were split into training drugs (or training target proteins) and test drugs (resp. test target proteins). The associated drug–target pairs in the benchmark data were then split into a training set and a test set. Note that drugs (resp. target proteins) in the test set did not overlap with those in the training set in block-wise cross-validation. In both types of cross-validation, we trained a predictive model only on the training set and evaluated prediction accuracy on the test set. Table 1 shows the averages and standard deviations of area under the ROC curve (AUC) and area under the precision–recall curve (AUPR) scores over three repetitions. Multiple data integration gave the highest AUC and AUPR scores. The performance measures in block-wise cross-validation tended to be lower than those in pair-wise cross-validation, implying that predicting all potential targets of drugs that have no known targets is more difficult than predicting missing targets (off-targets) of drugs with known targets (that have at least one known target protein).

**Table 1.** AUC scores and AUPR scores in the 3-fold cross-validation experiments

Cross-validation	Drug data	Target data	AUC ± S.D.	AUPR ± S.D.
Pair-wise	Chemical	Sequence	0.936 ± 0.010	0.579 ± 0.079
Pair-wise	Chemical	Domain	0.925 ± 0.095	0.355 ± 0.069
Pair-wise	Side effect	Sequence	0.922 ± 0.010	0.481 ± 0.087
Pair-wise	Side effect	Domain	0.903 ± 0.086	0.336 ± 0.072
Pair-wise	Integration	Integration	0.952 ± 0.006	0.593 ± 0.087
Block-wise	Chemical	Sequence	0.870 ± 0.004	0.485 ± 0.006
Block-wise	Chemical	Domain	0.843 ± 0.005	0.282 ± 0.001
Block-wise	Side effect	Sequence	0.847 ± 0.005	0.364 ± 0.016
Block-wise	Side effect	Domain	0.829 ± 0.001	0.237 ± 0.007
Block-wise	Integration	Integration	0.892 ± 0.001	0.507 ± 0.008

## CONCLUSIONS AND FUTURE DIRECTIONS

DINIES enables users to predict unknown parts of drug–target interaction networks on a genome-wide scale in the framework of supervised network inference. The algorithms for supervised network inference have been presented in previous publications (6,12,14), but this is the first paper presenting the web server. One of the advantages of the server is the flexibility of the input data, providing high potential to analyze drug–target interaction networks in various aspects. As an example, we showed an application using chemical structure and side effect data for drugs and amino acid sequence and domain composition data for proteins. However, users can input any other type of data as long as they are represented in the form of profile or similarity matrices, such as cellular phenotype profiles for drugs and gene expression profiles for proteins. DINIES is potentially useful for detailed analysis of drug–target interactions of interest to users in various applications.
